# Supplementary figures and images for: Brn3a controls the soma localization and axonal extension patterns of developing spinal dorsal horn neurons
Source: PLoS One. 2023 Sep 21;18(9):e0285295. doi: 10.1371/journal.pone.0285295 (PMC10513334; doi:10.1371/journal.pone.0285295)

# S1 Figure

A

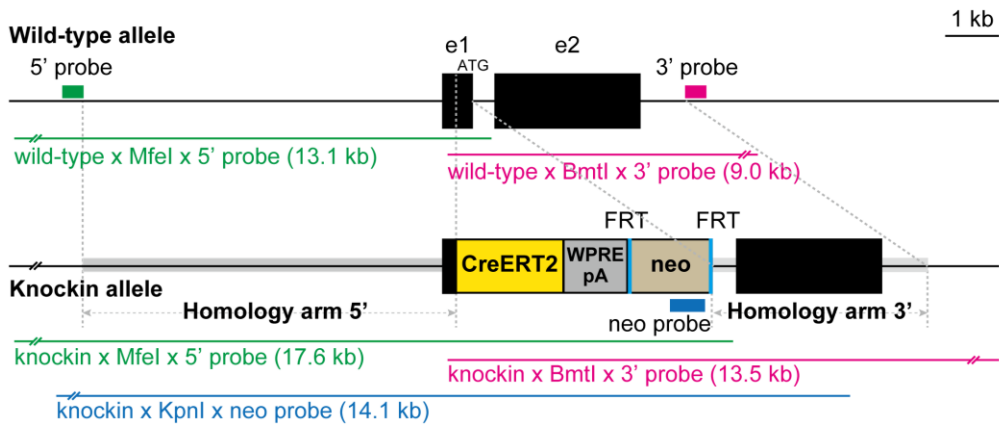

B

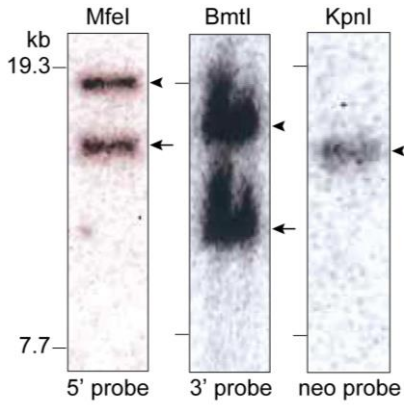

Supplement: S1 Fig — (A) Schematic representation of the wild-type and mutant Brn3b allele (Brn3bCreERT-Neo). The first exon of the Brn3b gene downstream of the translation initiation codon was replaced by a CreERT-FRT-Neo-ERT cassette to generate Brn3bCreERT-Neo allele. (B) Southern blot analysis of MfeI-, BmtI-, and KpnI-digested genomic DNA from Brn3bCreERT-Neo/+ ES cells. The 5’ probe identifies MfeI fragments of 13.1 kb (wild-type) and 17.6 kb (mutant). The 3’ probe identifies BmtI fragments of 9.0 kb (wild-type) and 13.5 kb (mutant). The neo probe identifies a KpnI fragment of 13.5 kb (mutant). Arrows and arrowheads indicate fragments derived from wild-type and mutant alleles, respectively. (PDF) [file pone.0285295.s001.pdf]

# S2 Figure

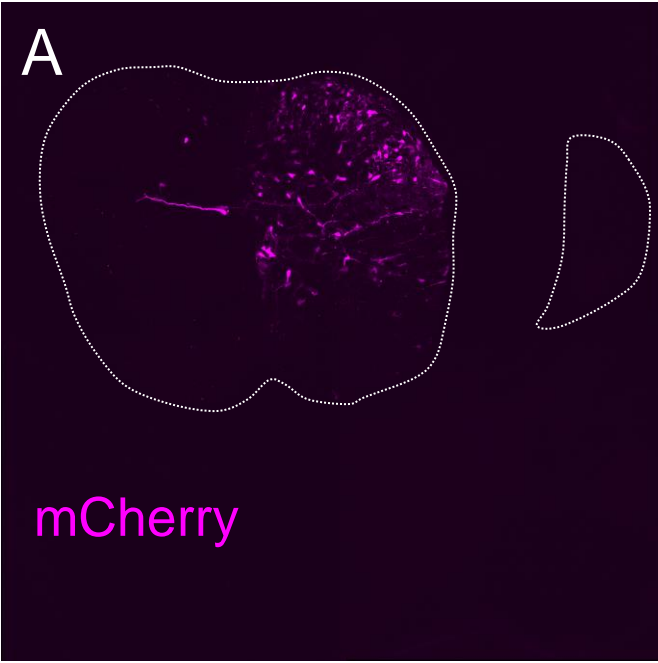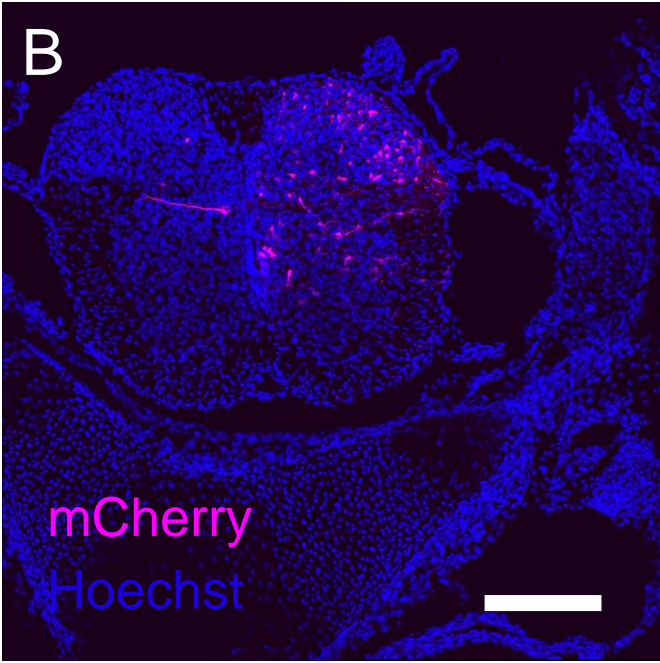

Supplement: S2 Fig — pCAG-mCherry was unilaterally introduced into spinal dorsal horn neurons of Brn3aCre/+ mice at E12.5 by in utero electroporation, and the spinal cord of the mice was dissected at E18.5. Fluorescence of mCherry (magenta; A, B) and Hoechst 33342 (blue; B) on the transverse section of the sample is shown. White dotted lines indicate the outline of the spinal cord and DRG. Note that mCherry-positive cells are only found in the spinal cord but not DRG. Scale, 200 μm. (PDF) [file pone.0285295.s002.pdf]

# S3 Figure

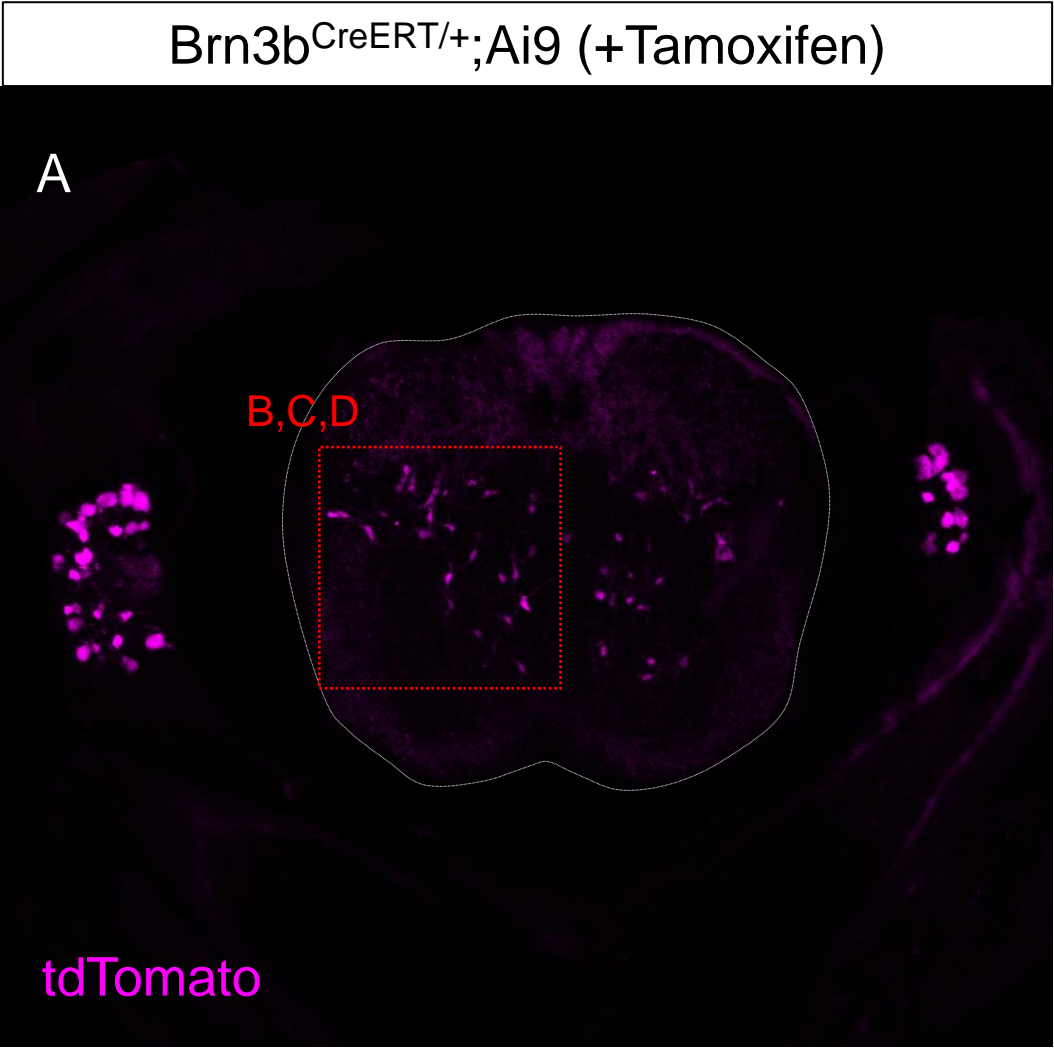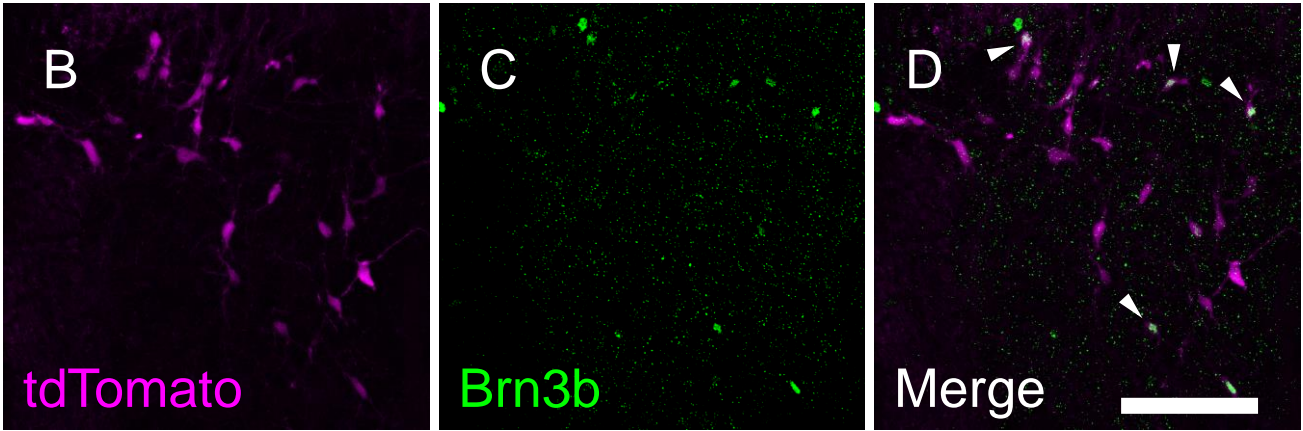

Supplement: S3 Fig — Tamoxifen was intraperitoneally injected into the pregnant Brn3bCreERT/+;Ai9 mice from E10.5 to E14.5 to label Brn3b-lineage spinal dorsal horn neurons. Transverse sections of the spinal cord of the mice at E18.5 were immunostained with anti-Brn3b antibody. (A) Fluorescence of tdTomato in the spinal cord of the mice is shown. White dotted line indicates the outline of the spinal cord. (B-D) High magnification views (marked in A) of tdTomato fluorescence (magenta; B, D) together with Anti-Brn3b immunostaining (green; C, D) are shown. Arrowheads in D indicate double-positive neurons. The percentage of Brn3b-positive neurons among tdTomato-positive ones was 43.4 ± 1.5% (447 cells [n = 4 mice]). Scale, 100 μm. (PDF) [file pone.0285295.s003.pdf]

# S4 Figure

A

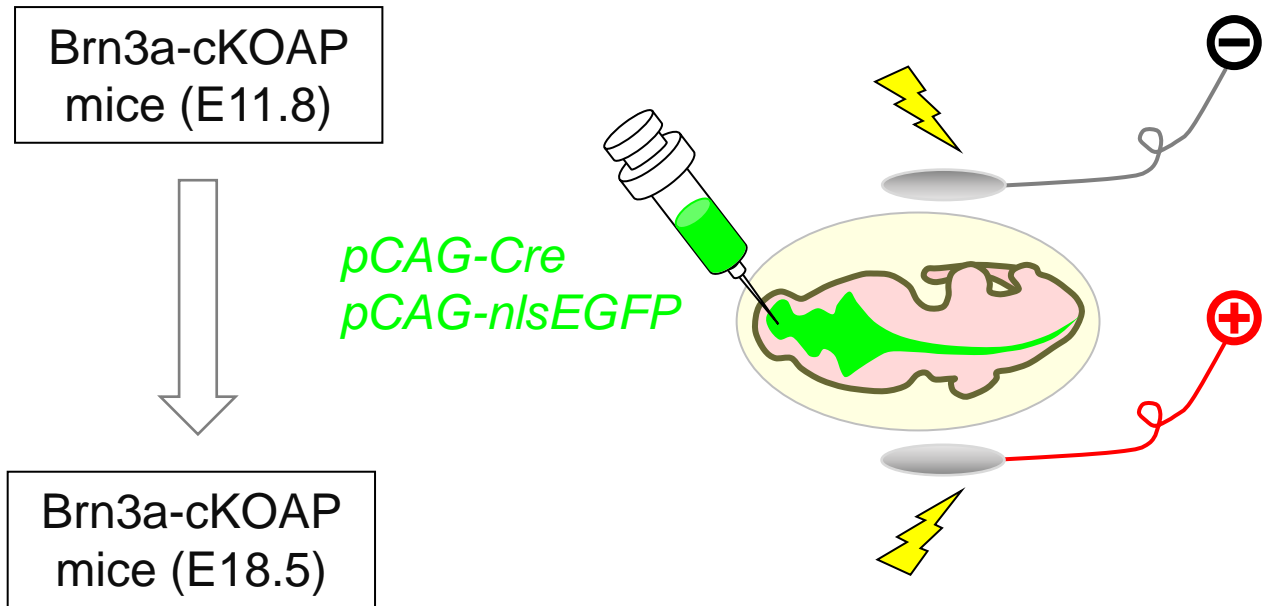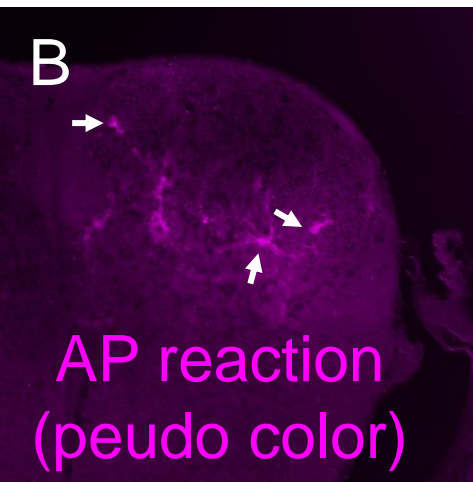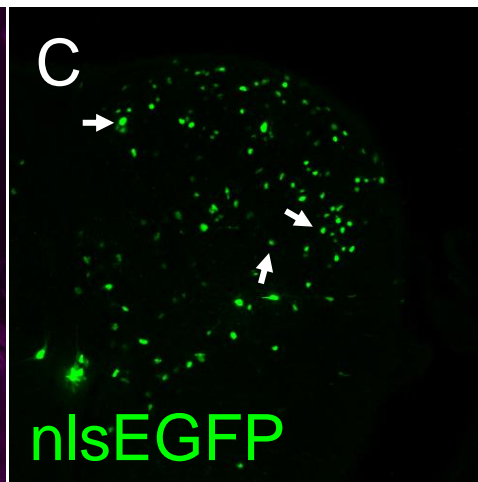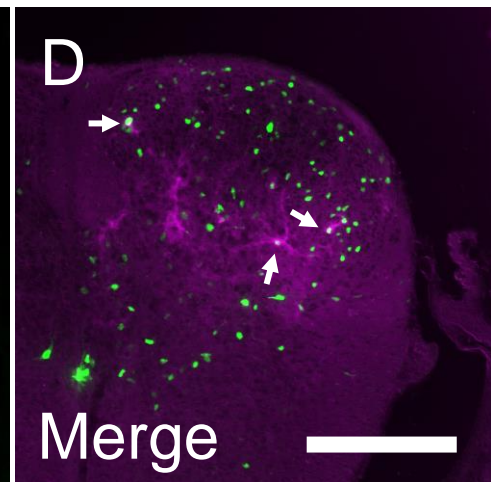

Supplement: S4 Fig — (A) pCAG-Cre together with pCAG-nlsEGFP were introduced into spinal dorsal horn neurons of Brn3a-cKOAP mice at E11.8 by in utero electroporation, and the spinal cord was dissected out at E18.5. AP assay was performed on the transverse section of the spinal cord to visualize the distribution of Brn3a-KO neurons. (B-D) AP signal (magenta; B, D) and nlsEGFP fluorescence (green; C, D) on the right spinal dorsal horn are shown. Arrows indicate double-positive neurons. Scale, 200 μm. (PDF) [file pone.0285295.s004.pdf]

# S5 Figure

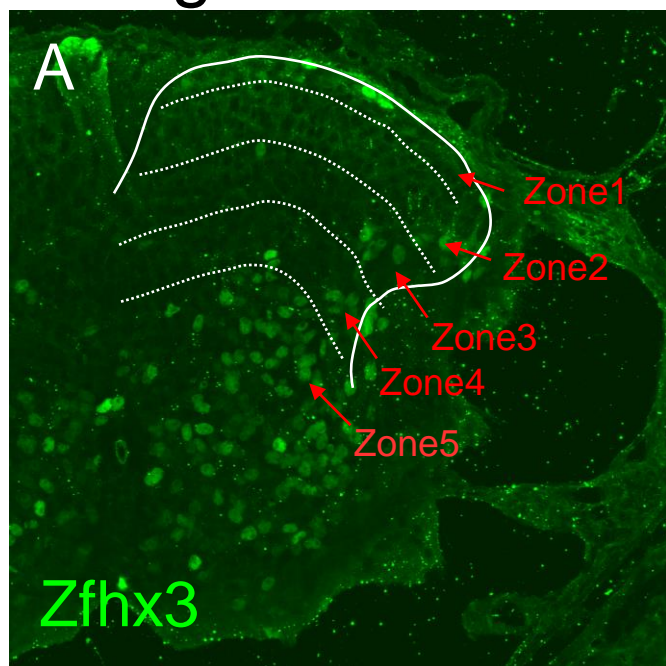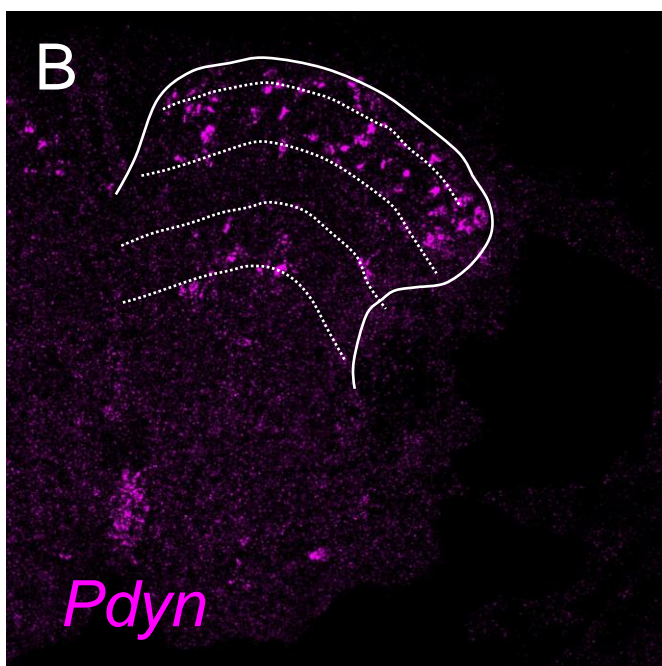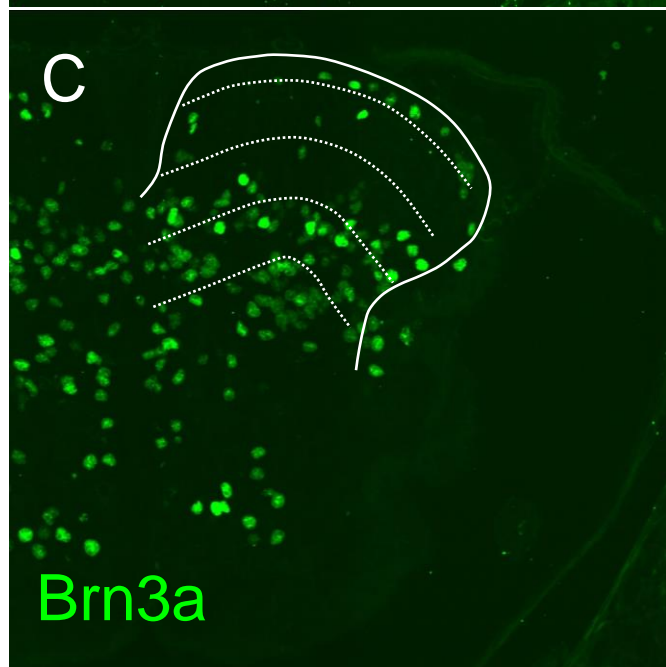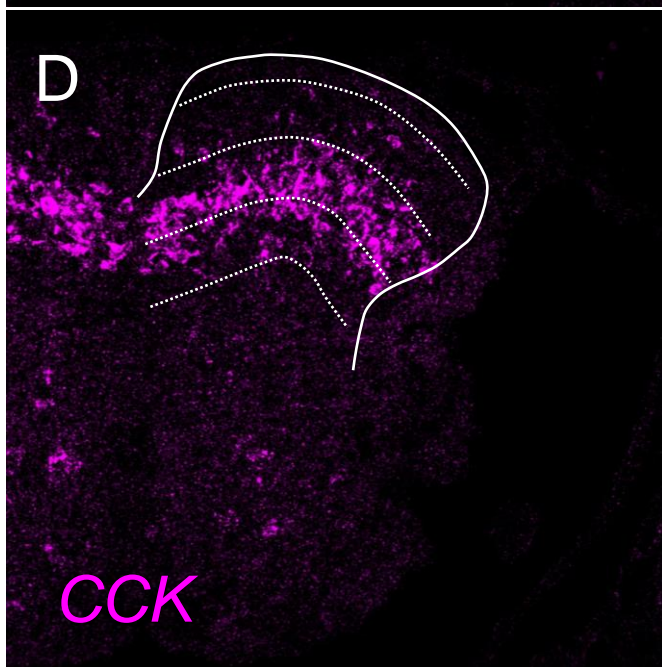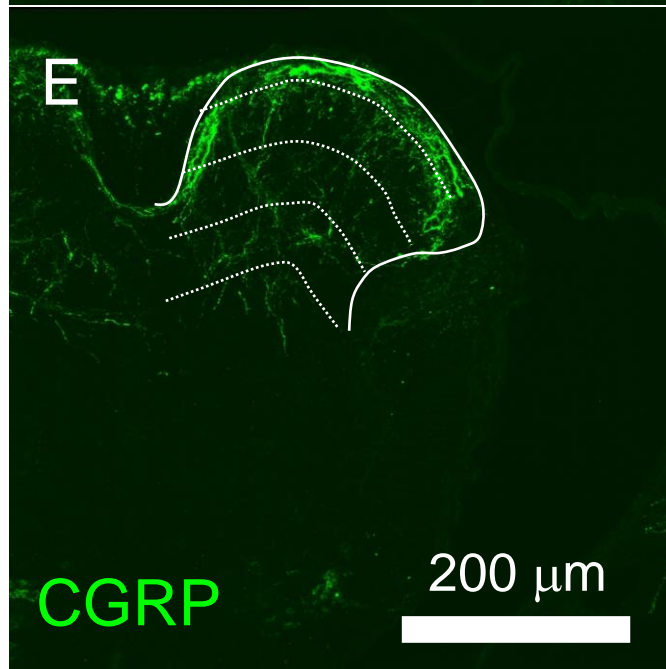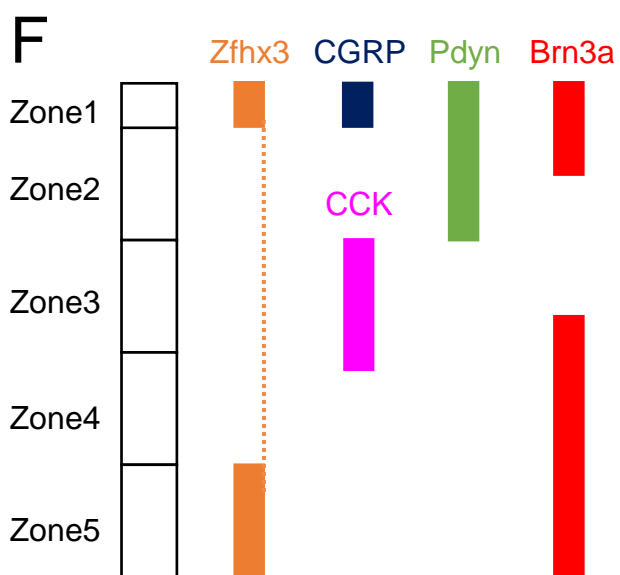

Supplement: S5 Fig — (A-E) Immunostaining of Zfhx3 (A), Brn3a (C), and CGRP (E), together with in situ hybridization of Pdyn (B) and CCK (D) were performed on the transverse section of the spinal cord of C57BL6J mice at E18.5. White solid lines indicate the boundary between gray and white matters. White dotted lines indicate the boundary of zones. (F) Schematic diagram of the expression pattern of marker molecules in each zone of the spinal dorsal horn at E18.5. Orange dotted line indicates the expression of Zfhx3 in the lateral edge of zone 2–4. (PDF) [file pone.0285295.s005.pdf]

# S6 Figure

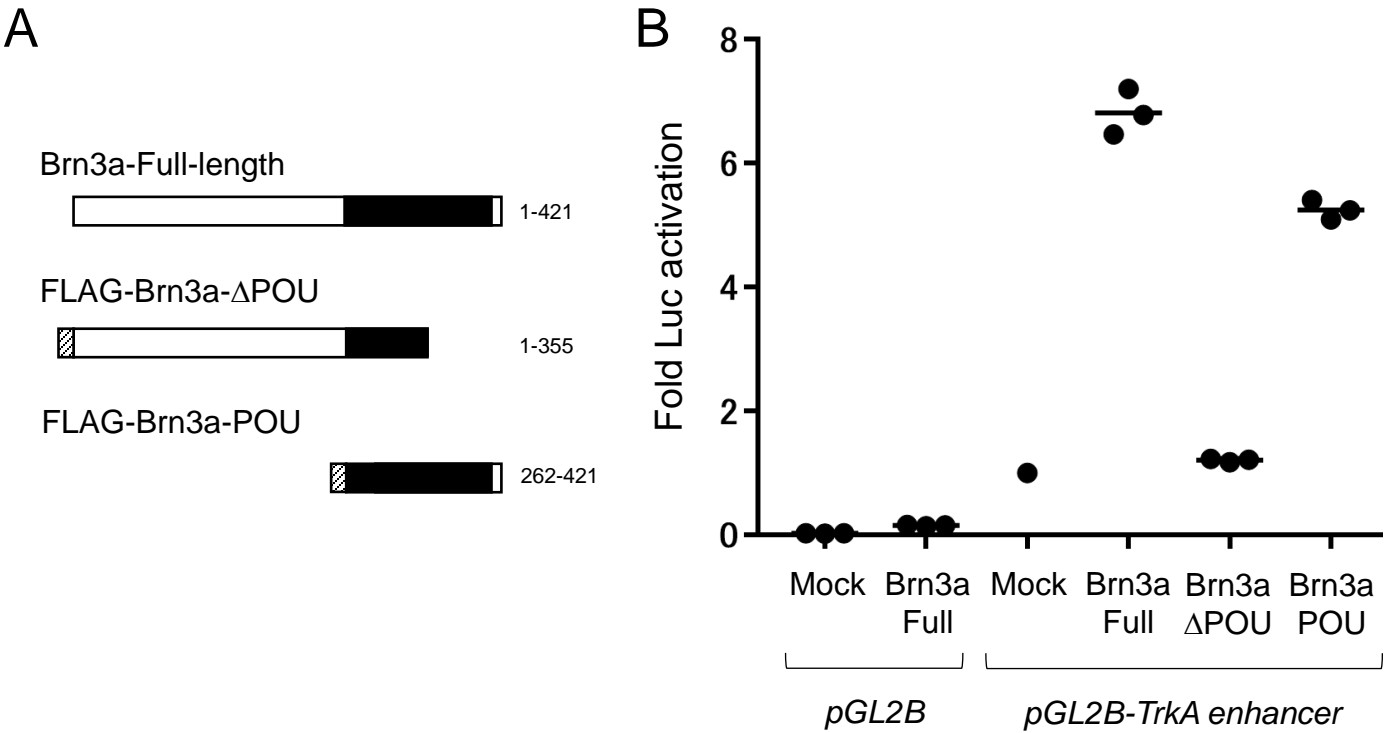

*nlsEGFP + FLAG-Brn3a-POU*

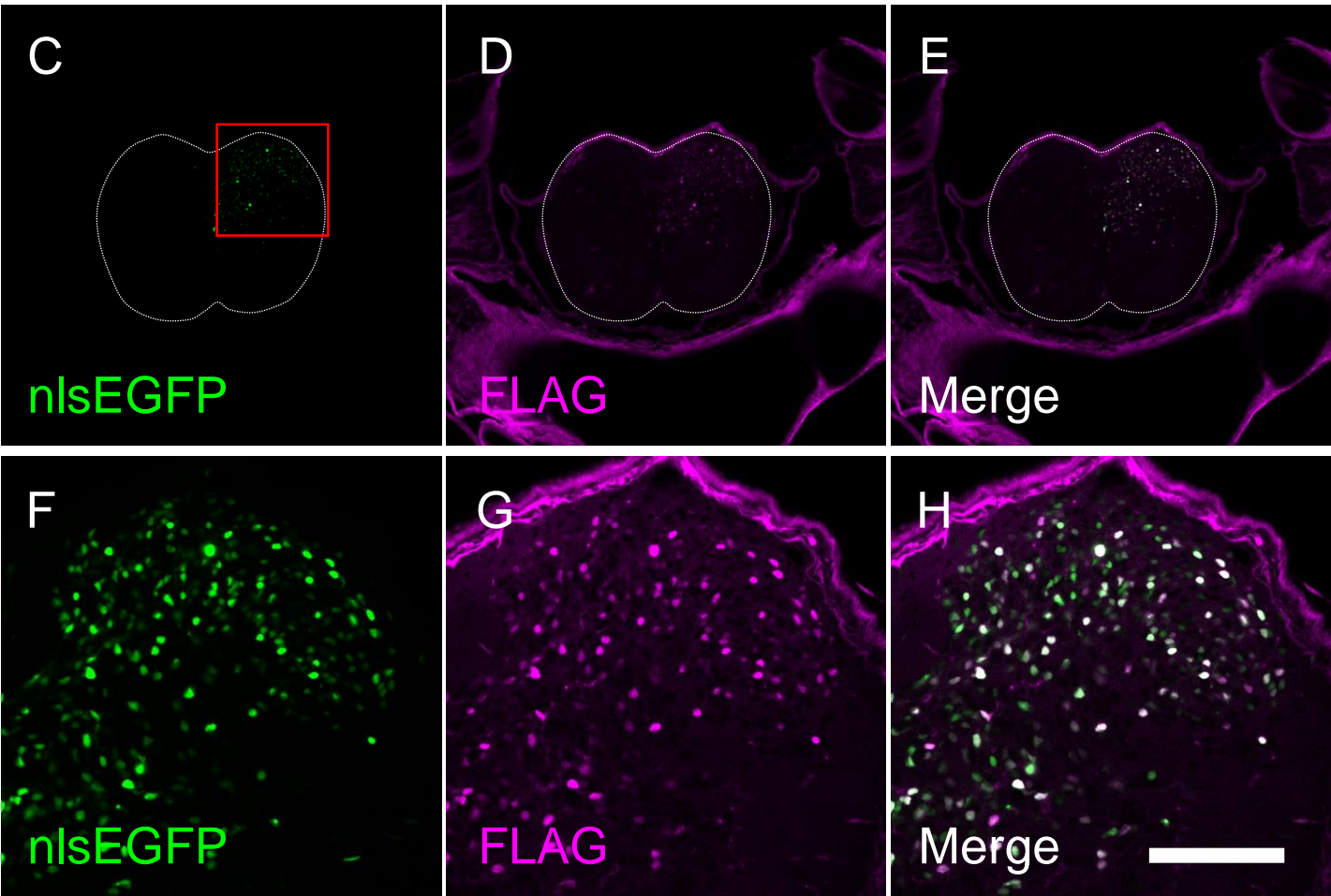

Supplement: S6 Fig — (A) Schematic diagram of full-length Brn3a and its truncation mutants. The black and hatched boxes indicate the POU homeobox domain and FLAG-tag, respectively. Mouse Brn3a contains 421 amino acids. FLAG-Brn3a-ΔPOU lacks C-terminal half of the POU homeodomain. FLAG-Brn3a-POU contains POU homeodomain and five C-terminal amino acids. (B) Transcriptional activities of Brn3a and its truncation mutants. pGL2B or pGL2B-Trk enhancer together with indicated Brn3a constructs were transfected into COS7 cells, and the luciferase activity of the lysate measured 2 days after transfection. The activity of firefly luciferase was normalized by that of co-transfected Renilla luciferase. Fold luciferase activity relative to the control sample (pGL2B plus pCAG) is shown (pGL2B, 0.03 ± 0.00; pGL2B+Brn3a-Full, 0.15 ± 0.01; pGL2B-TrkA+Brn3a-Full, 6.82 ± 0.21; pGL2B-TrkA+Brn3aΔPOU, 1.21 ± 0.01; pGL2B-TrkA+Brn3aPOU, 5.25 ± 0.09). Horizontal bars indicate the median. (C-H) FLAG-Brn3a-POU overexpressed in the spinal dorsal horn. nlsEGFP together with FLAG-Brn3a-POU were introduced into spinal dorsal horn neurons of the mice at E12.5 by in utero electroporation. The spinal cord of the mice was dissected at E18.5, and transverse sections of the mice were immunostained with the anti-FLAG antibody. Fluorescence images of nlsEGFP (green; C, E) and anti-FLAG immunostaining (magenta; D, E) are shown. White dotted lines indicate the outline of the spinal cord. High magnification images (marked in C) of nlsEGFP (green; F, H) and anti-FLAG immunostaining (magenta; G, H) are shown. Scale, 100 μm. (PDF) [file pone.0285295.s006.pdf]

# S7 Figure

A

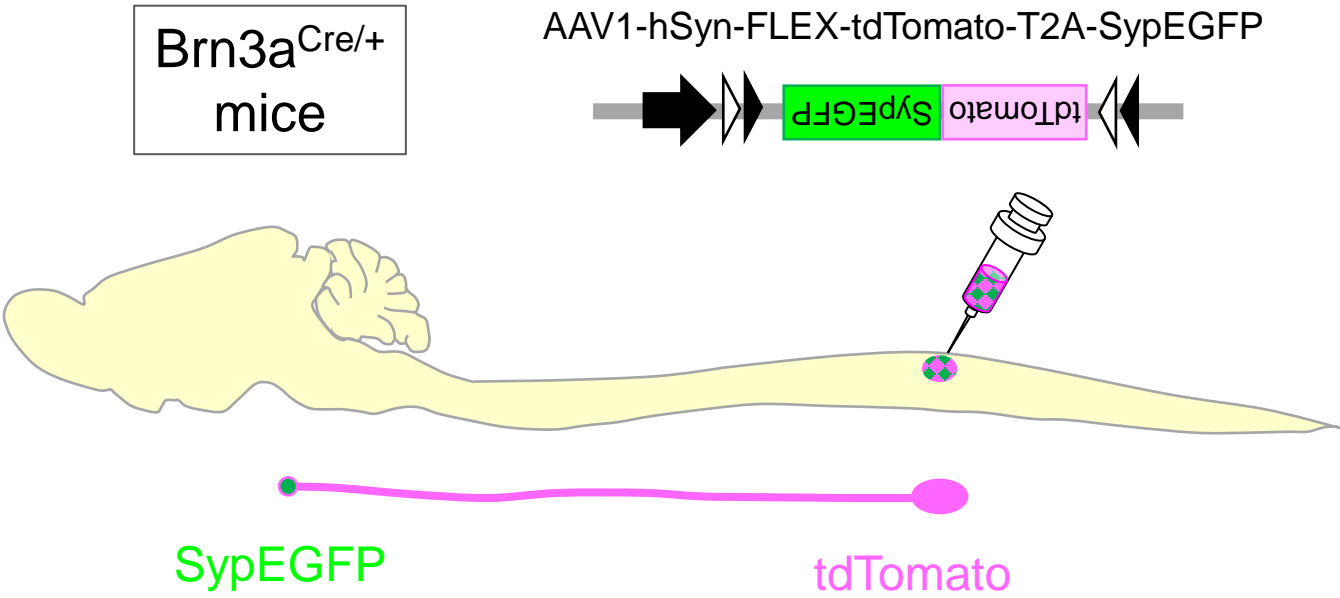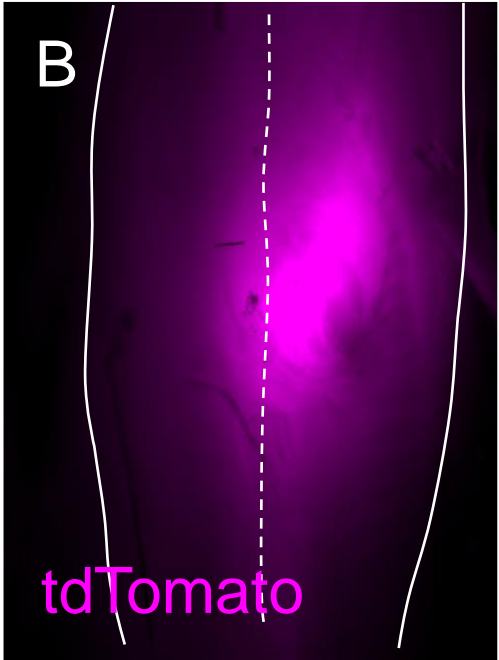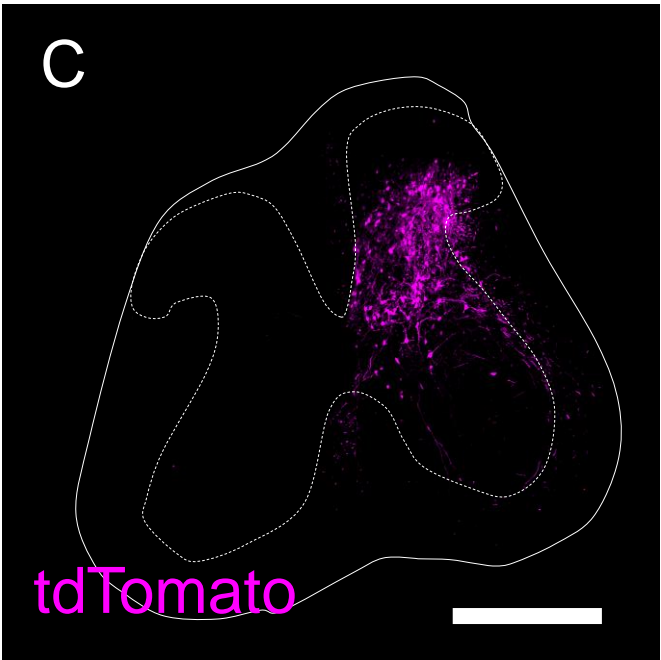

# S7 Figure

tdTomato

SypEGFP

D

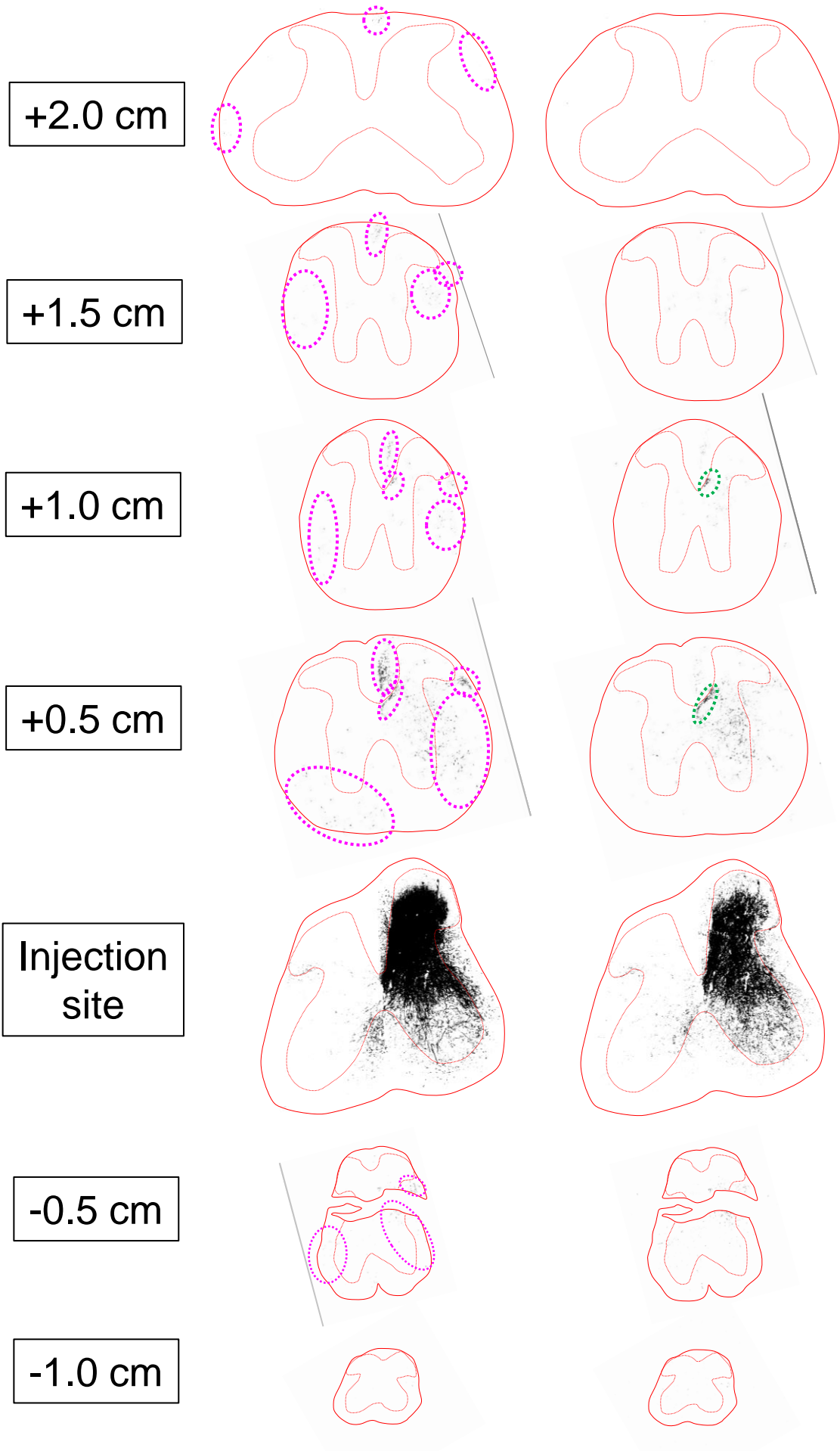

# S7 Figure

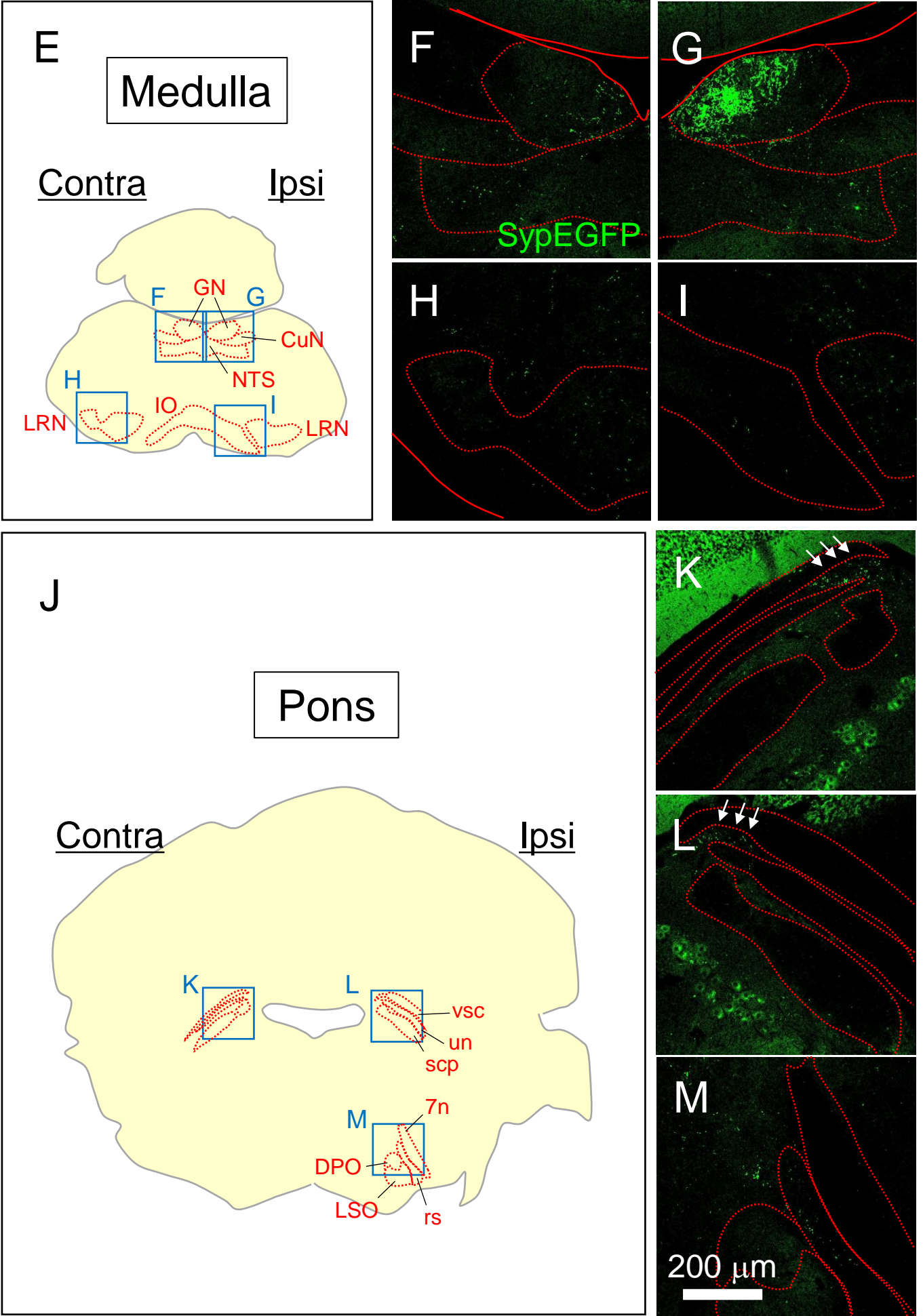

Supplement: S7 Fig — (A) AAV carrying FLEX-switched tdTomato and SypEGFP genes was injected into the lumbar spinal cord (~L5) of Brn3aCre/+ mice. This experiment enables labeling of Brn3a-persistent neurons and their presynaptic terminals by tdTomato and SypEGFP, respectively. (B-M) Three weeks after AAV injection, the spinal cord and whole brain of the mice were dissected out to analyze the distribution of axonal tracts and terminals of Brn3a-persistent neurons. tdTomato-positive cells were distributed in the lumbar spinal dorsal horn on the right. (B) Dorsal view of the spinal cord of AAV-injected mice. Solid and broken lines indicate the outline and the center of the spinal cord, respectively. (C) A transverse section of the lumbar spinal cord. A solid line indicates the outline of the spinal cord whereas a broken line indicates the boundary between the gray and white matters. Scale, 500 μm. (D) Distribution of tdTomato (left) and SypEGFP (right) fluorescence throughout the spinal cord of AAV-injected mouse. The area where tdTomato-positive cells were distributed was about 1.5 mm along the rostrocaudal axis. Fluorescence image of the representative transverse section in this area is shown in “injection site”. Fluorescence images rostrally (+2.0 cm, +1.5 cm, +1.0 cm, and +0.5 cm) and caudally (-0.5 cm and -1.0 cm) away from the injection site are also shown. The axonal tract labeled with tdTomato is distributed in the ipsilateral dorsal funiculus (DF), ipsilateral dorsolateral funiculus (DLF), ipsilateral ventrolateral funiculus (VLF), contralateral ventral funiculus (VF), contralateral VLF, or ipsilateral deep dorsal horn (circled by dotted magenta line). The fluorescence of tdTomato and SypEGFP is pseudocolored in black. The axonal tract on the contralateral side was mainly distributed in the VF near the injection site (+0.5 cm), but was found in the VLF on the section distant from the injection site (+2.0 cm, +1.5 cm, and +1.0 cm). Throughout the spinal cord, accumulation of presyn [file pone.0285295.s007.pdf]

# S8 Figure

A

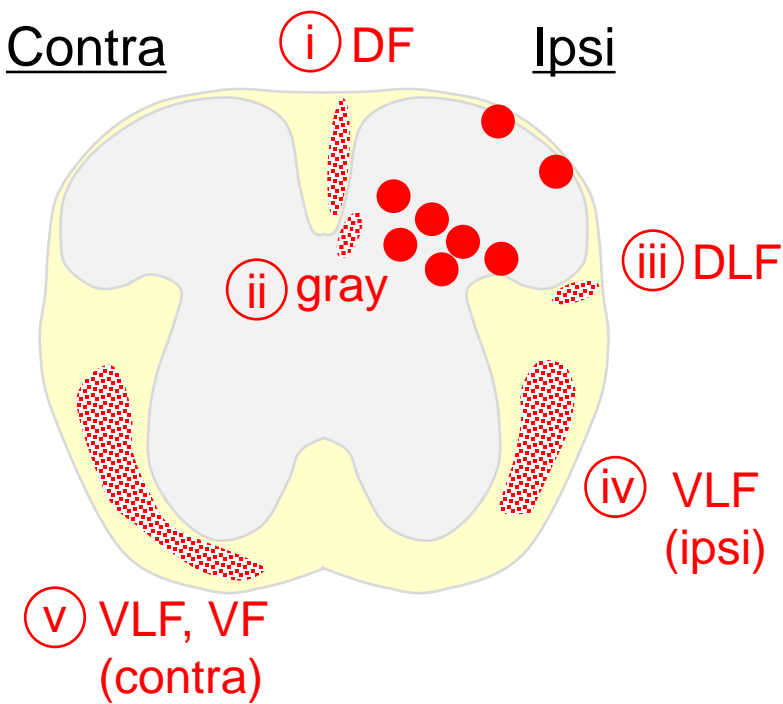

B

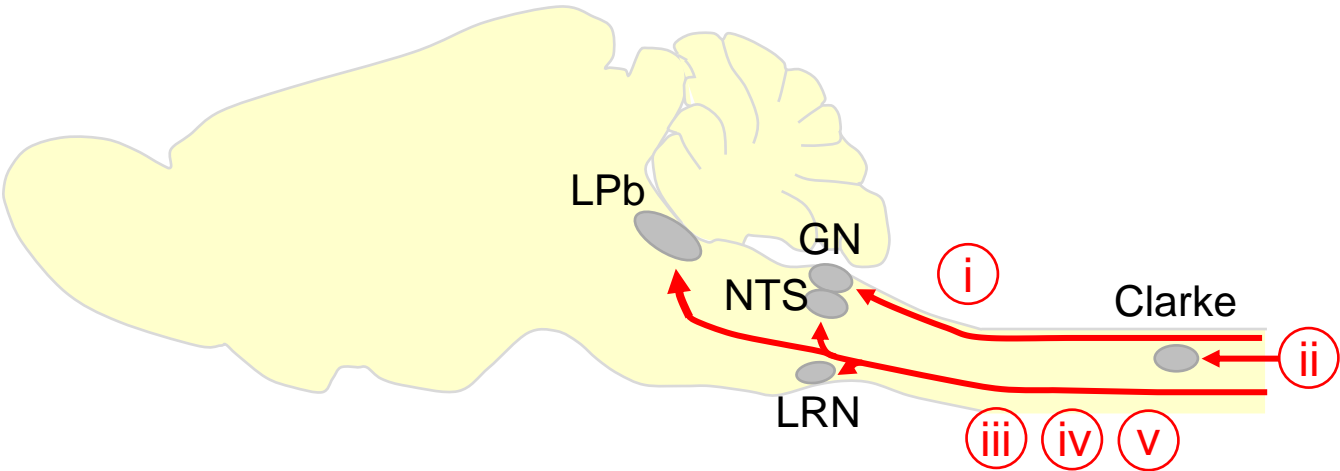

Supplement: S8 Fig — (A) Results in S8 Fig suggest that Brn3a-persistent neurons extend axons toward 5 different areas in the spinal cord: ipsilateral DF, ipsilateral dorsal horn, ipsilateral DLF, ipsilateral VLF and VF, and contralateral VLF and VF. The former 2 axons extend rostrally whereas the latter 3 axons do toward both rostral and caudal direction. (B) Previous anatomical studies suggest Brn3a-persistent neurons extending axons toward the ipsilateral DF innervate GN, whereas those extending axons toward LF and VF do several nuclei including NTS, LRN, or LPb. It is likely that Brn3a-persistent neurons extending axons toward ipsilateral deep dorsal horn innervate the Clarke nucleus. (PDF) [file pone.0285295.s008.pdf]

# S9 Figure

Cre  $\Rightarrow$  Brn3a<sup>cKOAP/+</sup>

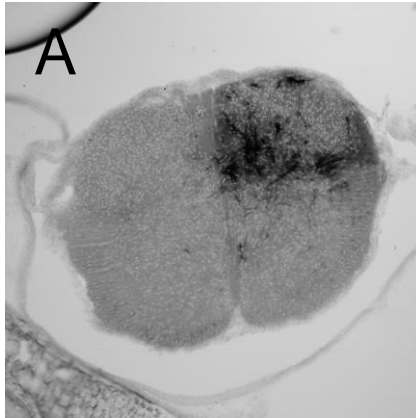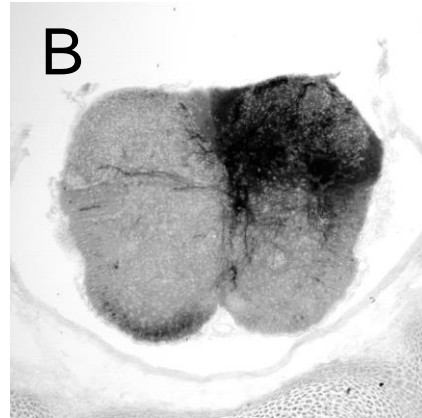

Cre  $\Rightarrow$  Brn3a<sup>cKOAP/cKOAP</sup>

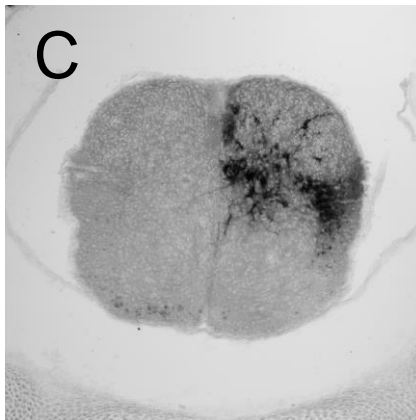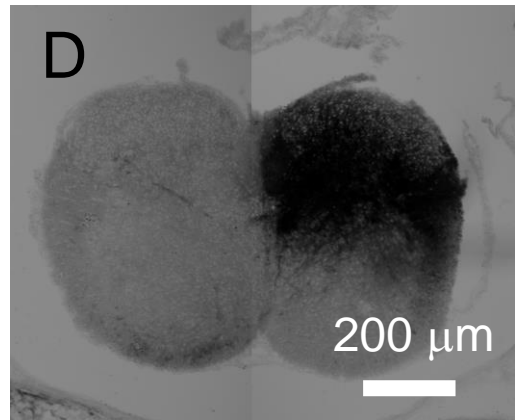

Supplement: S9 Fig — Axonal extension of Brn3a hetero and KO spinal dorsal neurons shown in Fig 3 was examined in the other mouse examples. AP staining images in the spinal cord of 2 Brn3acKOAP/+ (A, B) and 2 Brn3acKOAP/+ (C, D) mice are shown. Cre recombinase was introduced into the thoracic (A-C) and cervical (D) spinal cord on the right. Scale, 200 μm. (PDF) [file pone.0285295.s009.pdf]

# S10 Figure

A

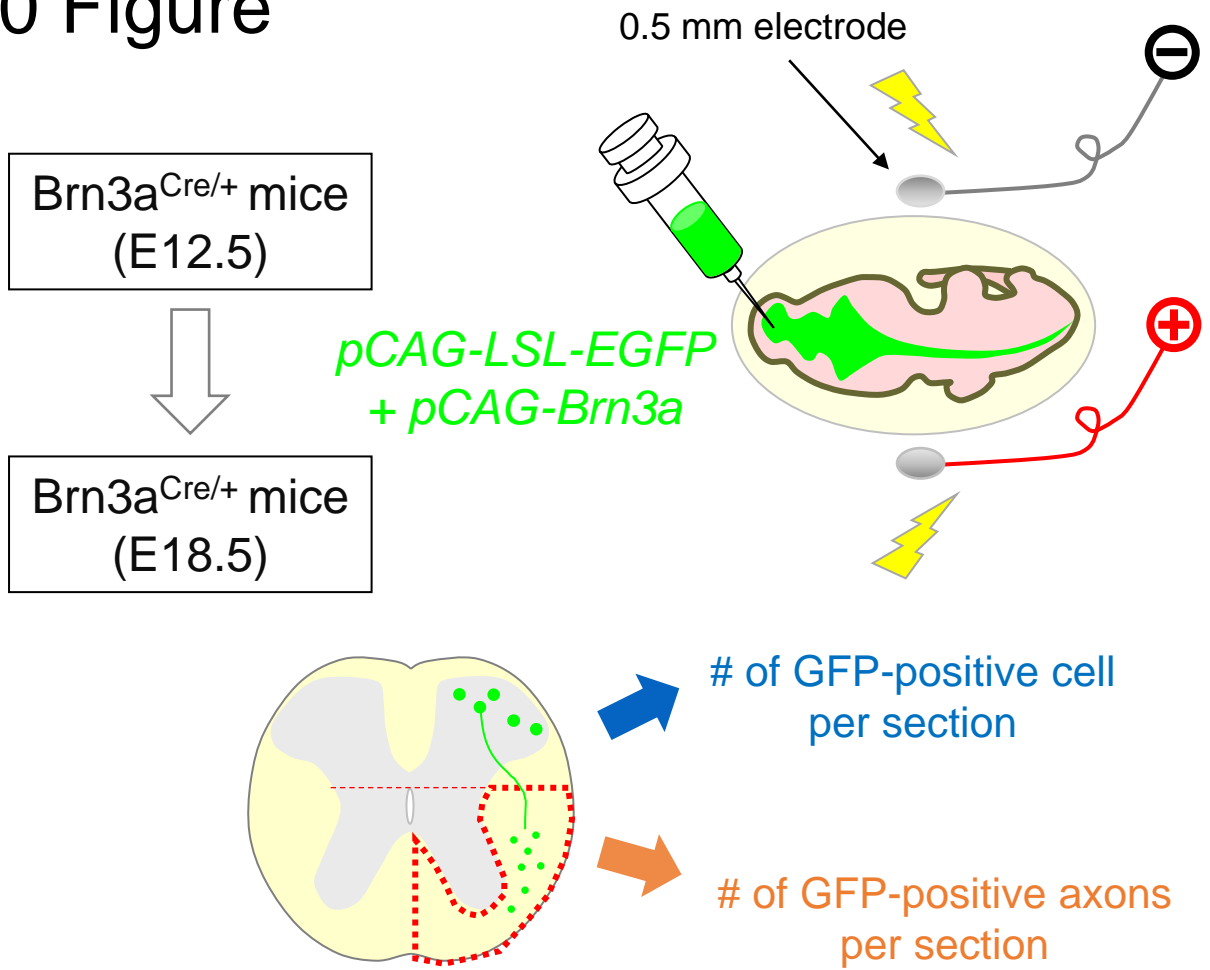

B

Mouse #4

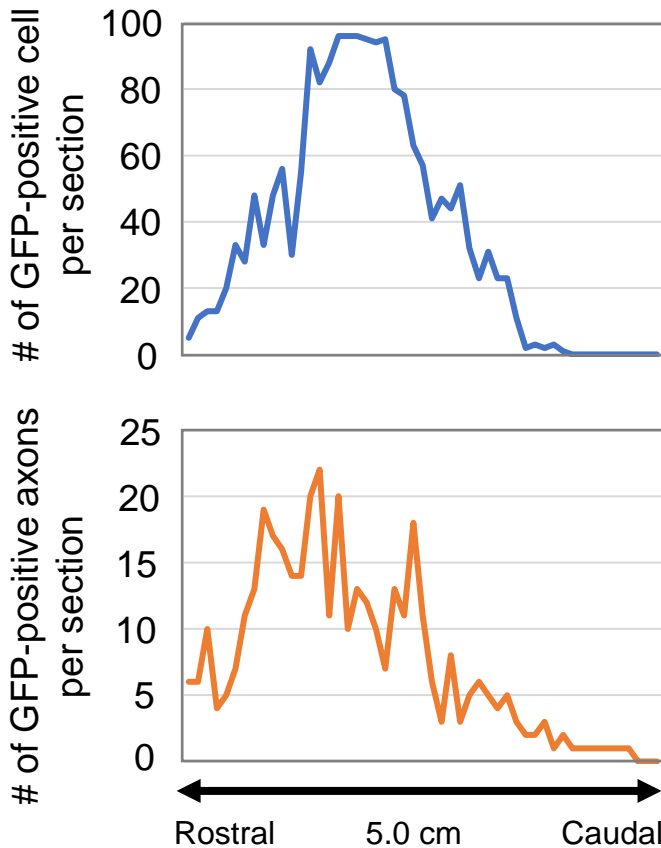

Mouse #7

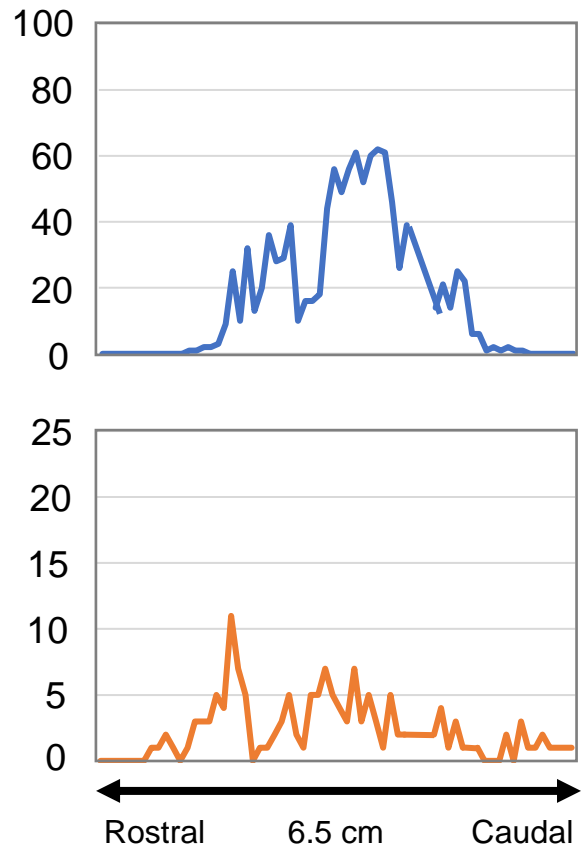

Supplement: S10 Fig — Rostrocaudal axonal extension of Brn3a-overexpressed neurons (A) pCAG-LSL-EGFP together with pCAG-Brn3a were focally introduced into spinal dorsal horn neurons of Brn3aCre/+ mice at E12.5 by in utero electroporation (0.5 mm round electrode). The spinal cord of the mice was dissected out at E18.5 to analyze the rostrocaudal axonal extension of Brn3a-overexpressed neurons. (B) The number of EGFP-positive cell bodies in the spinal dorsal horn as well as EGFP-positive axons in the ipsilateral VLF and VF was analyzed on the serial transverse sections of the spinal cord of 2 mice (mouse #4 and #7). The number of EGFP-positive cell bodies (blue) and EGFP-positive axons (orange) on each section is shown. The left and right sides of each graph indicate the sections located rostral and caudal to the electroporation sites, respectively. (PDF) [file pone.0285295.s010.pdf]

# S11 Figure

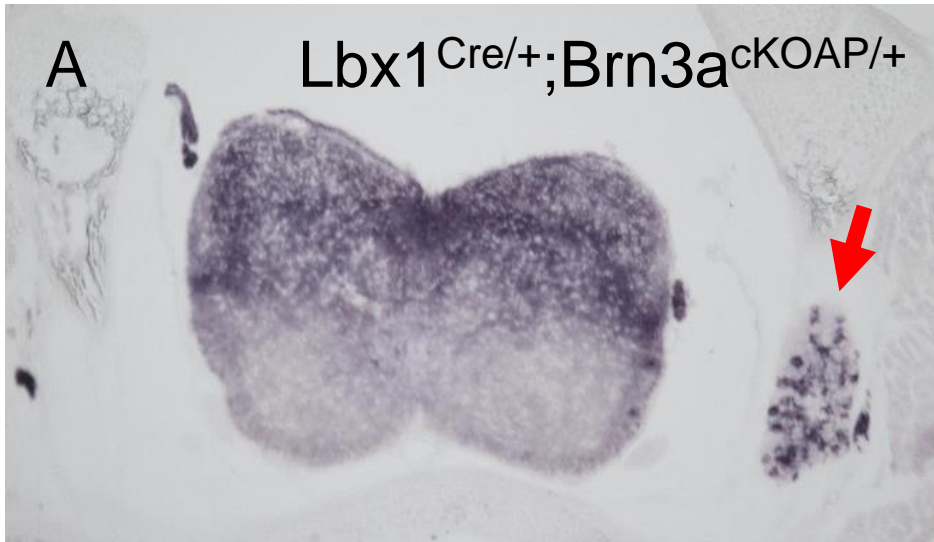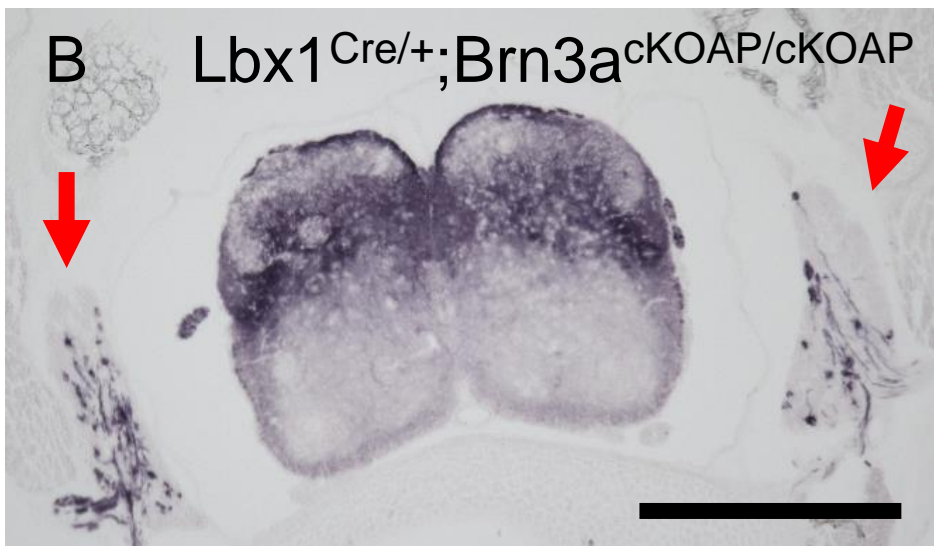

Supplement: S11 Fig — AP assay was performed on the transverse sections the spinal cord of Lbx1Cre/+;Brn3acKOAP/+ (A) and Lbx1Cre/+;Brn3acKOAP/cKOAP(B) at E18.5. Nonspecific recombination occurred in a population of DRG neurons (red arrows). Scale, 500 μm. (PDF) [file pone.0285295.s011.pdf]

S12 Figure

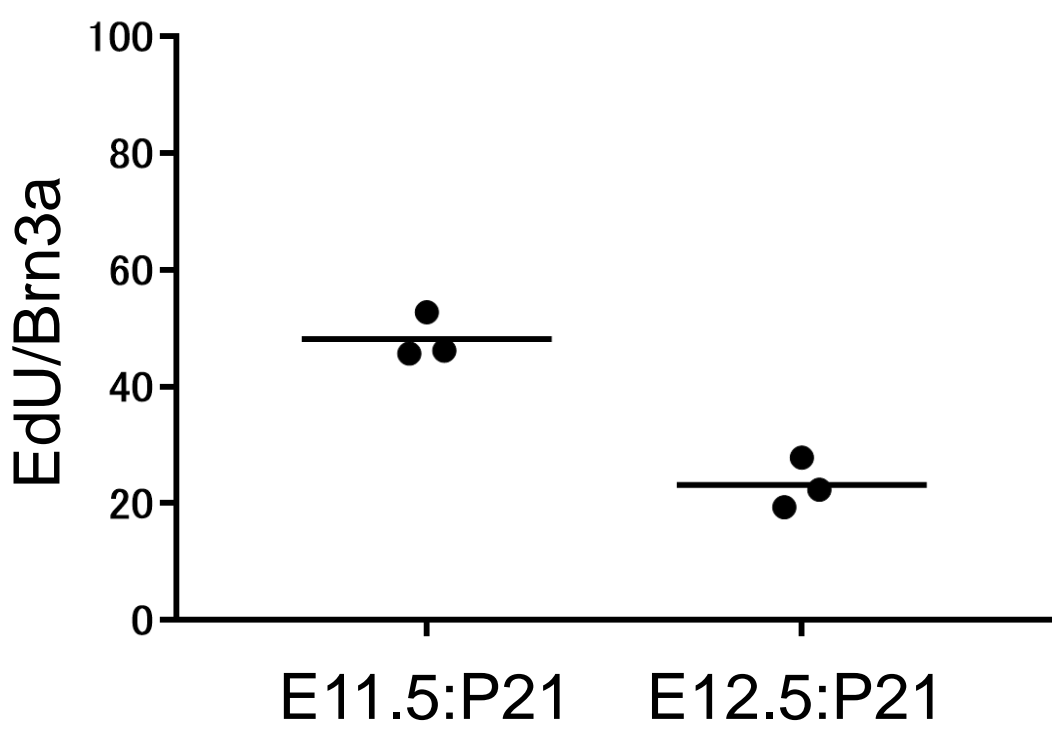

Supplement: S12 Fig — EdU was intraperitoneally injected into the pregnant C57BL/6 mice at E11.5 and E12.5, and the spinal cord of the mice was dissected at P21. EdU staining together with immunostaining with anti-Brn3a antibody were performed on the transverse sections of the thoracic spinal cord of the mice. The percentage of EdU-positive cells among Brn3a-persistent ones is shown (E11.5:P21, 48.2 ± 2.3%, 697 cells [n = 3 mice]; E12.5:P21, 48.2 ± 2.3%, 750 cells [n = 3 mice]). Horizontal bars indicate the median. (PDF) [file pone.0285295.s012.pdf]
